# The Experimental and Numerical Research for Plastic Working of Nickel Matrix Composite Coatings

**DOI:** 10.3390/ma13143177

**Published:** 2020-07-16

**Authors:** Tomasz Dyl, Robert Starosta, Dariusz Rydz, Bartosz Koczurkiewicz, Wioletta Kuśmierska-Matyszczak

**Affiliations:** 1 Department of Marine Maintenance, Faculty of Marine Engineering, Gdynia Maritime University, Morska Street 81-87, 81-225 Gdynia, Poland; r.starosta@wm.umg.edu.pl; 2 Faculty of Production Engineering and Materials Technology, Czestochowa University of Technology, Armii Krajowej Avenue 19, 42-201 Czestochowa, Poland; rydz.dariusz@wip.pcz.pl (D.R.); koczurkiewicz.bartosz@wip.pcz.pl (B.K.); 3 Faculty of Mechanical Engineering and Computer Science, Czestochowa University of Technology, Armii Krajowej Avenue 21, 42-201 Czestochowa, Poland; wiolettakusmierska@gmail.com

**Keywords:** numerical and experimental analysis, surface layer, alloy and composite coating, plastic working, burnishing

## Abstract

In the machine, metallurgical, and shipbuilding industries, steel products with alloy and composite coatings based on nickel may be used. It is expedient to improve the physicochemical properties of the surface layer of products as they have a significant roughness value after thermal spraying. It is therefore important to finish the layers applied by flame spraying, where machining is used for this purpose. However, it causes a loss of coating material, which is quite expensive. Therefore, in order to reduce costs and improve the quality of the surface layer, the finishing treatment of nickel-based coatings by means of plastic working is used. Two types of plastic working were proposed: rolling and burnishing. Numerical and experimental tests of the plastic processing of alloy coatings were carried out. The roughness of the coatings after rolling decreased to 1/25 and 30% strengthening of the alloy coating matrix was determined. After burnishing, roughness was reduced to 1/12 and the alloy coatings were strengthened by 25%. Plastic working by rolling and burnishing has a beneficial effect on the surface quality of the workpiece, not only by significantly improving the roughness, but also by increasing the strength properties of the surface layers.

## 1. Introduction

An important issue in theoretical considerations and experimental research of plastic working was to determine dependencies between technological parameters and the properties of the surface layer of materials. The close cooperation of designers with process engineers at the construction and technological stage of production preparation can significantly affect the improvement quality of manufactured products. Already at the design stage, it is necessary to specify the quality requirements for components used in the construction of machinery and equipment. Durability and reliability are the most important selection criteria. The production engineer should choose the most economical production methods that enable the product to be made according to technological quality requirements. The technological quality of machine parts depends on the machining methods used and the treatment parameters used. These parameters and methods produced under constant operating conditions have a significant impact on the durability and reliability of machine parts, sets, and ready devices.

The work carried out the theoretical and experimental analysis of the impact of burnishing parameters on the surface texture, potential, and operational properties of the surface layer of materials [1,2].

The alloy coatings of chromium are one of the solutions to extend the life of machines. However, due to the toxicity of the bath and the low current efficiency of the process of obtaining chromium galvanic coatings, alternative coating technologies and coating materials are sought. One of the proposals is the use of nickel and nickel matrix composite coatings obtained by various technologies (e.g., galvanic or spraying methods [1,2]). Composite coatings with a metal (nickel) matrix are technical coatings that increase the service life of machine parts in tribological nodes, or protective coatings. It is believed that by properly selecting the matrix material and reinforcing phase (e.g., ceramics), coatings have the most optimal performance properties possible [3,4,5,6]. Thermal spraying processes have become competitive with all of the known manufacturing and regeneration processes. The largest range of applications are the wearing parts of machines and devices. The use of spraying techniques allows for the reduction of manufacturing costs and an increase in the life of machinery and equipment. According to the authors [7,8], the process of thermal spraying has many advantages, which includes that the technology can be used in both unit and serial production, is a quick method of producing layers with the required properties, devices are relatively simple, the range of coating materials is very wide, and thermal spraying allows for layers to be obtained from all groups of engineering materials (metals and their alloys, polymers, ceramics-oxides, carbides, nitrides, metal borides, and composites). Nickel alloys are resistant to gas and electrochemical corrosion, hence the industry’s great interest in coatings made of this material for protective layers. Nickel-aluminum alloy coatings are considered to be materials that are resistant to general corrosion in seawater [9,10,11]. Improvements in the performance of machine parts including pump shafts can be achieved by using coatings. Coatings obtained by thermal spraying achieve high surface roughness values. Therefore, it is required that the thermally sprayed coatings be applied, taking into account the finishing allowance. Finishing should not only ensure the appropriate thickness of the coatings related to the nominal size of the object, but also to obtain the required surface roughness and corrugation. Most often for this purpose, machining is used, less often grinding. Machining is used for heat sprayed coatings with a thickness exceeding 1 mm [1,4,12].

In this work, to apply regenerative coatings, due to the technological susceptibility of the ship’s engine room workshop and repair shipyards, the methods of flame and plasma spraying were selected. ProXon 21021 powder containing nickel and 5% aluminum were chosen as the coating material. Despite the relatively low hardness, it is recommended for the regeneration of machine parts working in tribological connections. nickel-based alloy can be operated in conditions that may cause chemical and electrochemical corrosion. Ni–5%Al alloys are considered as corrosion resistant in a seawater environment. Strengthening of coatings based on ProXon 21021 material may occur as a result of incorporating a reinforcing phase into the structure in the form of dispersion ceramic particles. MetaCeram 28020 material consisting of aluminum oxide particles was used as reinforcement. Aluminum oxide is resistant to corrosion and tribological wear, and is characterized by a low coefficient of friction [12].

It is important to provide machine components with appropriate technological quality while maintaining the requirements for mechanical and operational properties. Therefore, it is significant to the finishing treatment of steel surface layers by applying coatings by flame spraying of alloy and composite materials. Coatings applied by flame spraying have a high roughness, therefore they must be subjected to a finishing operation [13,14,15,16,17]. The application of methods based on plastic deformation is increasing among those for the finishing of machine parts. The burnishing process for alloy and composite heat sprayed coatings can be used instead of machining. Burnishing is a plastic surface treatment carried out on cutting machines as finishing and strengthening machining. The burnishing process does not produce chips or sparks, as in the case of using chip and abrasive machining. For this reason, burnishing is currently known as one of the ecological treatment methods [18,19,20,21,22,23].

## 2. Material Selection and Scope of Research

The nickel-aluminum alloy coatings Ni–5%Al (ProXon 21021; 93.45% Ni; 5% Al; 0.8% B; 0.34% Fe; 0.18% Cr; 0.15% Si; 0.08% C) and nickel-based composite coatings Ni–5%Al–15%Al_2_O_3_ (ProXon 21021 and MetaCeram 28020; 97.6% Al_2_O_3_; 2.2% TiO_2_; 0.2% SiO_2_, products by Castolin Eutectic, Lausanne, Switzerland) were applied to samples of C45 unalloyed steel (Table 1) by thermal spraying material powder, where the percentage share of mass carried out was nickel 93.45% and aluminum 5%, and the composite coating was an additional 15% Al_2_O_3_ (products by Castolin Eutectic [12], Lausanne, Switzerland). Flame spraying of alloy coatings was carried out with the following technological parameters: inflammable gas pressure–acetylene: 0.07 MPa; oxygen pressure: 0.4 MPa; distance between blowpipe and sprayed surface: 150 mm; number of applied layers: 12; and the obtained coating thicknesses were 0.6 mm–1.2 mm. The plasma sprayed alloy and composite coatings were fabricated by Plasma System S. A. from Siemianowice Slaskie, Poland. The following parameters of the plasma thermal spraying process were used: argon flow 2000 dm^3^/h; hydrogen flow 100 dm^3^/h; distance between burner blowpipe and machined surface 100 mm; current intensity 450 A–600 A; and arc voltage 47 V–60 V.

Computer simulations of the rolling and burnishing process were designed to visualize the phenomena occurring during the formation of the surface layer. The state of stress and the state of deformation in the surface layer were analyzed. This issue is particularly important because it provides the opportunity to choose optimal parameters of plastic forming of the surface layer. A coating of nickel and aluminum alloy by flame spraying was used on flat rolled steel products. The results of the research on the influence of the quantity of plastic strain on the consolidation and stereometric properties of the surface layer are detailed in [13,14].

The theoretical analysis allows us to state that the most even replacement strain distribution was obtained in an alloy coating 0.6 mm thick for 5% relative draft. The research results of applying the thin coating process and using low draft values are included in [15,16,17]. On their basis, it could be concluded that it is possible to use the surface plastic working of Ni–5%Al alloy coatings that have been thermally sprayed.

The first stage of simulation design was model creation. In the model, the STL grid (STereoLithography, FORGE^®^, Transvalor S.A., Sophia–Antipolis, France) was applied. The program allowed for the accurate application of a finite element grid to the designed materials. An objective of the rolling process computer simulation was to obtain the flow curve both base (steel C45) and nickel-based coating materials. This research was carried out with a dilatometer–plastometer DIL 805A/D (BAHR Thermoanalyse GmbH, Hüllhorst, Germany). The commercial packet based on the finite element method was applied in the computer simulations. The use of a computer program, which is based on a finite element method and has built-in thermo-mechanical models, requires the boundary conditions to be defined. The following input data for the numerical analysis was determined: the initial temperature was 20 °C; the heat exchange coefficient between the workpiece and the tool was 3000 W/Kmm^2^; and the heat exchange coefficient between the material and the air was 100 W/Kmm^2^. The coefficient of the sliding friction of steel was 0.1.

The objective of the research was to determine the optimal thickness and behavior of the coating in the area of deformation. In the work, the state of deformation and stress in the material the nickel-base alloy coating were analyzed after cold working.

The dependence yield stress of strain intensity, strain rate, and temperature used for the numerical research can be approximated by the Hensel–Spittel [24] formula expressed as:

(1) $$\sigma_{p}=K_{0}\;e^{m_{1}T}\;\varepsilon^{m_{2}}\;{\dot{\varepsilon }}_{i}^{m_{3}}\;e^{\frac{m_{4}}{\varepsilon }}$$

where *σ_p_* is the yield stress; *T* is the temperature; *K*_0_, *m*_1_, *m*_2_, *m*_3_, and *m*_4_ are the coefficients of the function (*K*_0_ = 1521 MPa, *m*_1_ = −0.003, *m*_2_ = −0.127, *m*_3_ *=* 0.145, *m*_4_ = −0.059 for the C45 unalloyed steel; *K*_0_ = 596 MPa, *m*_1_ = −0.0009, *m*_2_ = 0.2625 for Ni–5%Al alloy coating); *ε* is the strain intensity; and $\dot{\varepsilon_{i}{}_{\;}}$ is the strain rate intensity.

Equation (1) is characteristic of the numerical analysis because it represents the connection between stress and strain. The coefficients given in the formula are defined on the basis of dilatometric–plastometric studies. Coefficient values were determined for the core material and coating materials.

Flat steel samples with 0.6 mm thick coating were prepared for testing. The cold rolling was performed for two relative deformations of 5% and 10% at a constant rolling speed 0.2 m/s. The corrosion tests performed at the same university showed that samples with the Ni–5%Al alloy coating, rolled with higher reduction values, did not show a decrease in the corrosion properties. For lower deformation ratios, an improvement in corrosion resistance was reported. Two type of duo rolling mills were used: ϕ150 mm on a 170 mm barrel length, and ϕ200 mm on a 250 mm barrel length.

Burnishing was executed by means of a NK-01 disc burnisher (Department of Marine Maintenance, Gdynia, Poland). During burnishing, the following parameters were applied: feed *p* = 0.044 mm/rev–0.8 mm/rev; burnisher pass depth a_n_ = 0.2 mm–0.3 mm; burnishing force *F* = 0.7 kN–1.1 kN; burnishing speed v_n_ = 28 m/min; and burnishing element with a diameter of ϕ50 mm.

Research on the influence of coating plastic working on the value of internal stress was carried out. Diffraction records were made on a Bruker D8 Discover x-ray diffractometer (Billerica, MA, USA).

The stereometric parameters of the surface layer before and after the plastic working were measured on the samples using a Hommel-Etamic T8000 profilometer (The Sempre Group Ltd., Gloucester, UK) in accordance with EN ISO 4287:1997 [25] where many of the surface stereometric parameters were measured. The hardness testing machine FM-800 was used, which enabled precise and semi-automatic measurements according to the Vickers method (HV). Hardness and micro-hardness measurements were conducted by the Vickers method according to EN ISO 6507-1:2018 [26]. The microstructure of the coatings (porosity) was assessed by quantitative metallography based on graphical models of thermally sprayed coatings. Microstructure investigations were performed using an Axio Observer D1 MAT optical microscope (ZEISS, Oberkochen, Germany).

## 3. Numerical Investigations and Analysis of Plastic Working

In model considerations of plastic working by burnishing, the reaction of tools on an object is often limited to [13,16,22,23] the reaction of the tool on a small surface area of the contact with a deformed object, as described by Bussinesq solutions. It is a model of elastic–plastic half space loading with concentrated force; the reaction of a stiff tool with a specific curvature on a deformable object is known as the Hertz model. This model is a modification of the Bussinesq model and takes into account the fact that the burnishing force is distributed over a certain contact surface of the spherical tool with the workpiece.

**Figure 1:**
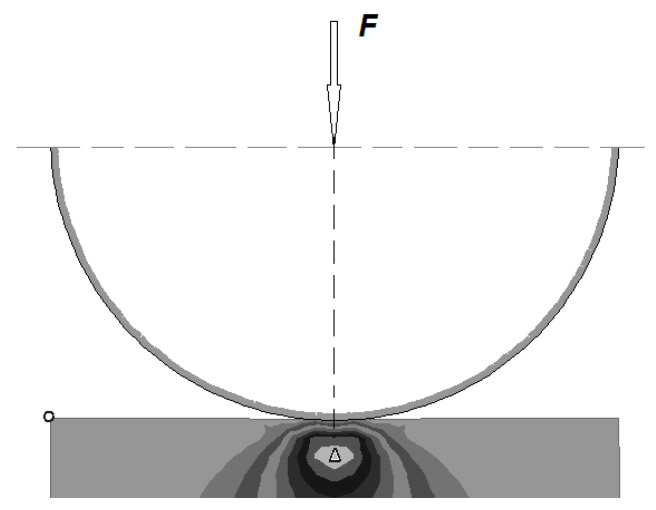
Scheme of realizing the contact of a rigid tool with a specified curvature on a deformable object.

In the burnishing process, the rigid burnishing element has a small radius of curvature in relation to the radius of curvature of the workpiece. The reaction of the tool on the object can be replaced by the interactions of force focused on half space. The concentrated contact of the spherical tool with the workpiece is constant (Figure 1).

The stress occurring at the contact point of two elements pressed together with maximum force reaches the highest values in the middle of the contact surface and are called nominal contact stresses, which are different to maximum pressure stresses. In relation to the elaboration in [13,16,27,28,29] on the definition of a numerical model of elastic–plastic surface plastic working, a number of theoretical analyses were conducted using the finite element method to determine the state of stress and strain at the contact point of the two pressed items. On the contact surface of the collaborating elements, it is possible to determine the state of stress and strain using the FORGE^®^ package based on the finite element method [13,30].

Determining the state of stress on the surface layer is a particularly important issue due to the possibility of predicting the mechanical state of machine elements with appropriate technological parameters. The surface plastic working increases the energy state of the surface layer [31,32,33], and thus affects the achievement of appropriate changes in the physical properties of the material in the surface layer, which reduces its abrasive wear and increases fatigue strength.

### 3.1. Results of Numerical Investigations and Analysis of Rolling

The analysis of the rolling process was performed on flat products of C45 unalloyed steel with a Ni–5%Al alloy coating by the flame spraying method. The analysis determined the intensity and stress intensity distributions in a Ni–5%Al alloy coating applied on a C45 unalloyed steel. As a result of the computer simulations of rolling the flat band with a coating thickness of 0.6 mm and 0.9 mm, the effect of draft on the uniformity of strain distribution was not observed. The increase in total strain value caused an increase in strain value in the Ni–5%Al alloy coating as well as in the C45 unalloyed steel bases. Larger strain gradients were due to the lower deformation resistance in the coating.

**Figure 2:**
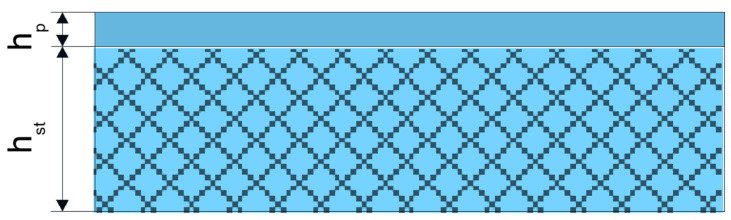
Scheme of the layer band layout prepared for the cold working process where h_p_ is the thickness of the coating and h_st_ is the height of the steel base.

The initial thickness of the rolled band was h_0_ = 10 mm. The ratios of the thickness of the layers assumed for the research were: h_st_/h_p_ = 9.4/0.6; 9.1/0.9; and 8.8/1.2 (where h_st_ is the thickness layer of C45 steel, and h_p_ is the thickness of Ni–5%Al alloy coating). In the plastic working process, the coating was in the top part of the band (Figure 2).

**Figure 3:**
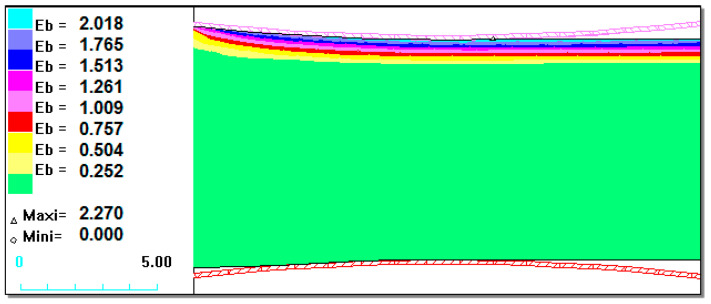
State of strain intensity for the Ni–5%Al alloy coating for 5% relative draft with a different coating thicknesses at the upper part rolling band of h_p_ = 0.6 mm.

**Figure 4:**
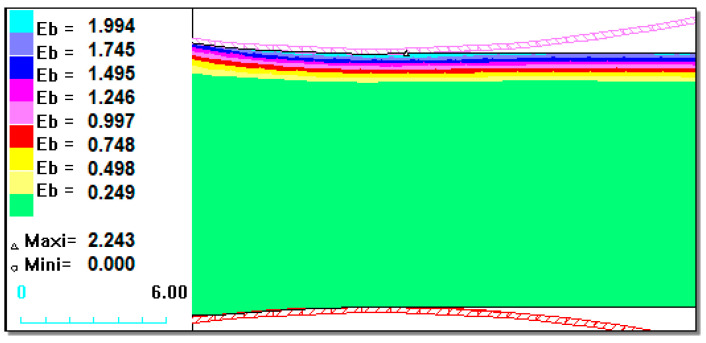
State of strain intensity for the Ni–5%Al alloy coating for 5% relative draft with different coating thicknesses at the upper part rolling band h_p_ = 0.9 mm.

**Figure 5:**
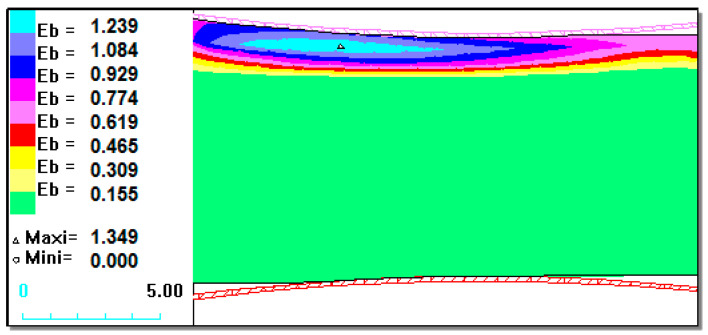
State of strain intensity for the Ni–5%Al alloy coating for 5% relative draft with different coating thicknesses at the upper part rolling band h_p_ = 1.2 mm.

Figure 3, Figure 4 and Figure 5 show that for the 1.2 mm thickness coating, a different state of strain was observed than for the 0.6 mm and 0.9 mm coatings. The maximal values of the replacement strain for smaller coating thicknesses (Ni–5%Al) occurred directly at the contact of the material with a roller, whereas for a 1.2 mm thickness coating (Figure 5), it occurred under the contact surface at the Bielajew’s point [16,33,34,35,36,37,38]. This distribution of strain intensity can cause possible delamination as result of loss of cohesion of the coating material or loss of coating adhesion in the actual rolling process. On the basis of the computer simulations carried out, we could certify that with the application of small values of relative draft (5%) and for a coating thickness of 0.6 mm (Figure 3), there was a uniform distribution of strains, which should provide good adhesion of the coating to the base during plastic working [13,39]. This has been confirmed in experimental studies. During the rolling process and already after its completion, the Ni–5%Al alloy coating was characterized by very good adhesion to the base. This was confirmed by qualitative methods of coating adhesion tests, which showed that rolling did not remove their adhesion to the steel base.

**Figure 6:**
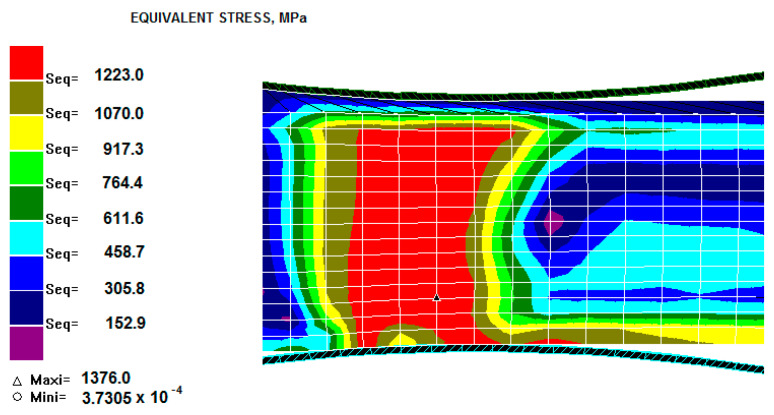
State of equivalent stress for the Ni–5%Al alloy coating for 5% relative draft with different coating thicknesses at the upper part rolling band h_p_ = 0.6 mm.

Figure 6 presents the distribution of equivalent stresses in the rolled strip zone from the deformation area.

Based on the data presented in Figure 3 and Figure 6, it can be concluded that the steel material in the core had the highest equivalent stress values and the lowest deformation values. As the coating thickness increased, the deformation value decreased. The equivalent stresses in the middle of the steel layer reached maximum values, which can be explained by a larger deformation of the coating. As a result, there was an increase in the additional tensile stress occurring in the steel.

### 3.2. Numerical Results of Modeling by Burnishing

**Figure 7:**
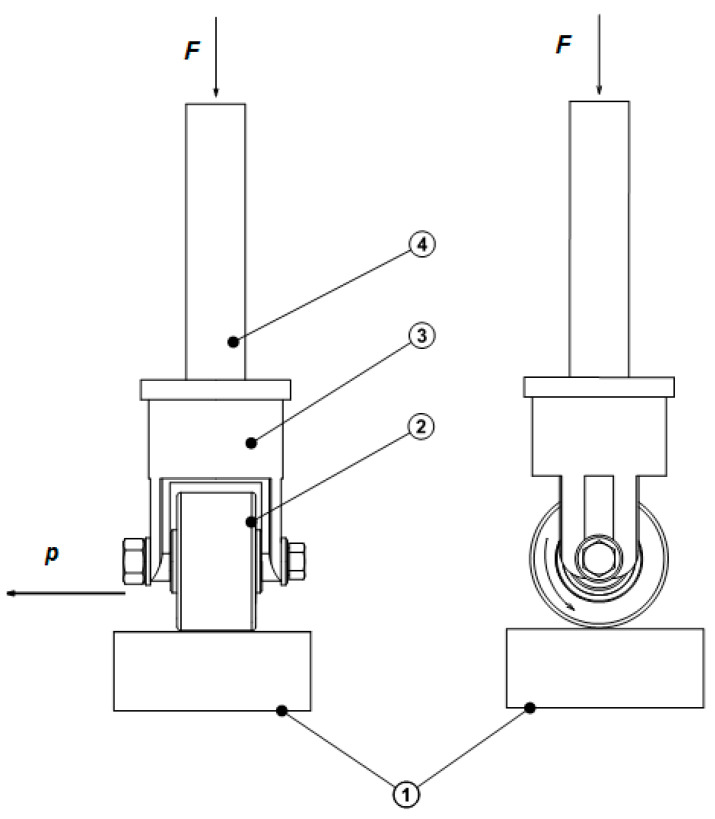
Scheme of the flat surface finish rolling process, where *F* is the burnishing force, *p* is the burnishing feed. (1) workpiece, (2) burnishing roller, (3) burnishing tool post, (4) clamp rigid.

The next stage concerning the modeling of the surface plastic working processes was to conduct a numerical analysis of pressure burnishing by finishing rolling nickel-based alloy coatings according to the scheme in Figure 7 [13,16,17].

After the computer simulations were realized, the state of the strain and stress for the C45 steel with the Ni–5%Al alloy coating was defined.

**Figure 8:**
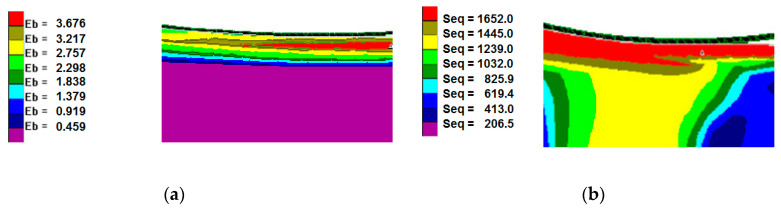
State of strain and stress in the Ni–5%Al coating after the burnishing process for a 0.6 mm coating thickness: (**a**) strain intensity; (**b**) stress intensity in MPa.

Figure 8 shows examples of the computer simulation results of the burnishing process for a 0.6 mm thickness coating.

The maximum values of strain intensity, stress intensity, and steady-stress component occurred at the contact of the working tool with the coating.

It can be seen in Figure 8 that the nickel-based coating occurred at the most intense state of strain, whereas in the steel substrate, the strain was minimum. After the computer simulations, it was determined that it is possible to deform the Ni–5%Al alloy coating by static turning into strain burnishing.

The analysis carried out on the cold rolling process found that the most advantageous distribution of strains and stresses was obtained for a thickness band coating of 0.6 mm. This is why in the burnishing process, such a value of the coating thickness was also proposed. Increasing the coating thickness caused a disadvantageous distribution of the strain and reduced stress, which could have led to delamination of the band. For economic and technological reasons, it is justified to apply coatings with the smallest thickness.

**Figure 9:**
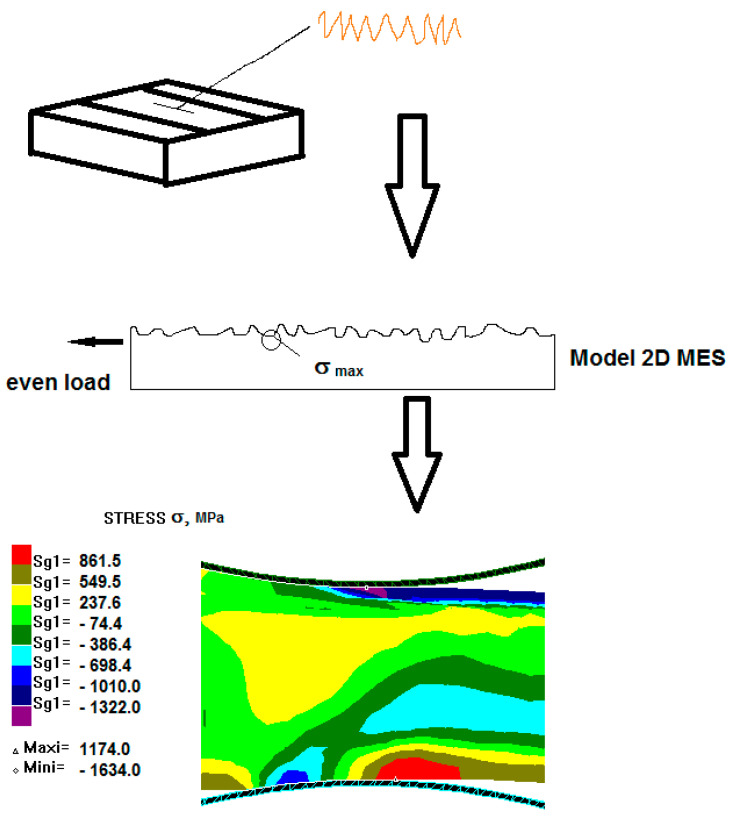
The principle of numerical calculations of stress concentration.

In the presented work, numerical analysis was a design stage of surface layer shaping. The aim of the research was to present the influence of rolling and burnishing on the surface. Based on the literature data [13,40], it can be stated that rolling and burnishing have an effect on the reduction of surface roughness (Figure 9).

The numerical tests carried out in the paper showed the dependence of stresses arising during rolling and burnishing on the deformations caused, affecting both hardening of the surface layer and reduction of the surface roughness.

## 4. Experimental Research of Plastic Working

According to the demand for improving the durability in the fatigue of machine components with a complex shape that are exposed to changing loads, a proper solution will be burnishing and rolling. This is most often used in machines and devices concerning machine-building, metallurgical, the shipbuilding industry, etc. Their fatigue strength can be increased by plastic working (rolling and burnishing), and the durability depends on many factors. Not only do the selection of appropriate constructional materials and the right concept of execution have an impact on the life cycle of the device, but the quality of the surface layer of machine components also performs a significant role. The required surface quality of machine elements is influenced by the technology used during their manufacture, and the quality of the surface layer is one of the most important factors determining the utility value of a product.

Burnishing also allows a surface characterized by small values of parameters determined from the material contribution curve to be obtained. In connection with the planned use of coatings to cooperate with the packing sealing, it is important that the burnishing surface achieves the condition of leak tightness. Due to the decrease in roughness and surface strengthening undoubtedly, there may be an increase in resistance to corrosion in a sea water environment, contact fatigue, and tribological wear as well as reducing the coefficient of friction and obtaining a flat characteristic of wear intensity compared to turned and ground coatings.

### 4.1. Experimental Research for Rolling

Experimental tests after the rolling process of flat products determined the influence of the quantity of strain on the reduction index of roughness of nickel-aluminum alloy coatings. The experimental and numerical analysis of the rolling process for producing plates, plane, ball, shaft, and round surface are presented in [13,14,15,16,41,42,43,44]. During the rolling process, the quality of the coating produced on the base material was analyzed as well as the values of permanent deformations for the applied relative drafts of ε = 5% and 10%. Based on the results of the experimental tests, the results of the numerical tests were verified. The conducted experimental verification shows that the statistical error between the obtained deformations in the modeling process and in the laboratory conditions ranged from 2% to 4%. It can be assumed that computer simulations correctly reflect the course of the actual formation of surface layers. A result of the experimental tests of the average value of main stress in the axial direction after surface plastic working was 243 MPa; the numerical tests showed that the average value was 256 MPa of the main stress in the axial direction, which means that the error was about 5%.

The nickel-aluminum rolled alloy coating had a considerably lower roughness compared to the surface roughness of Ra = 13.3 μm after thermal spraying. The arithmetic mean of ordinates of the surface roughness profile of the Ni–5%Al coating obtained values after turning of Ra = 1.95 μm and after grinding of Ra = 0.29 μm.

During abrasive machining coatings, bonded tools require frequent correction of their shapes so we performed the necessity of sharpening the grinding wheel working surface. Maintaining the cutting ability and appropriately shape the working surface of bonded tools was not easy to realize, therefore the grinding wheels wore out rapidly, and as a result, the machined surface coating was reduced in quality.

Thus, plastic working can be admitted as a more favorable alternative to the abrasive machining of alloy coatings. After cold rolling on a duo rolling mill with a roller diameter of ϕ150 mm, it turned out that the surface roughness of the Ni–5%Al coatings was Ra = 0.49 μm for the 10% draft and Ra = 0.52 μm for the 5% draft. For cold-rolled samples on rolling mill with a roller diameter of ϕ200 mm, the coatings showed less roughness (Ra = 0.28 µm for 10% relative draft, Ra = 0.33 μm for 5% relative draft) when compared with the surface roughness profile obtained after thermal spraying (Table 2). The indicator of the decrease in the roughness of coatings after plastic working K_Ra_ took values from around 25.

**Figure 10:**
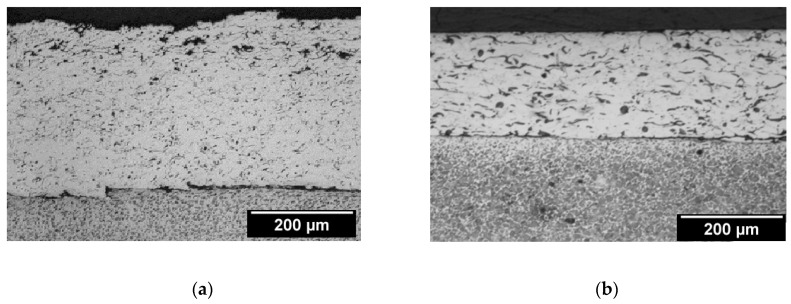
Microstructure of Ni–5%Al alloy coating, etched with 4% HNO_3_. (**a**) After flame spraying, the roughness of the coating was Ra = 13.3 μm. (**b**) After cold rolling (ϕ200 mm) for a relative draft of 10%, the roughness of coating was Ra = 0.28 μm.

The microstructure of a nickel-aluminum alloy cold-rolled coating is shown in Figure 10. It can be seen that the structure of the coating was homogeneous, mostly of closed pores formed during thermal spraying. The flame sprayed alloy and composite coatings were porous (Figure 10a). Metallographic studies showed a reduction in the porosity of plastically deformed coatings (Figure 10b). The estimated porosity after spraying in the alloy coatings was 17%. In composite coatings, it was higher and amounted to 21%. After rolling, the pores were closed. After plastic working, the porosity was obtained for the 5% alloy coatings and for the 9% composite coatings.

After rolling the steel samples with an alloy coating, there was a permanent deformation of the coating material. The carrier material was not observed to have had changes in the structure on its plastic deformation. In laboratory tests of rolling flat samples with an alloy coating, it was found that the deformation significantly improved the surface quality of the Ni–5%Al alloy coating. It was also found that thin coatings and low deformation values were justified. Research on the impact of the amount of plastic strain on the strengthening and stereometric properties of the surface layer of rolled flat steel manufacture with a nickel-aluminum alloy coating applied by flame spraying showed that it is possible to use plastic working as a finishing, in return for machining (chip and abrasive) thermally sprayed nickel alloy coatings. Thermal spray coatings based on nickel can be used, for example, to regenerate shafts of sea water pumps. However, as a finishing treatment of these coatings, burnishing can be used [4,13,17].

### 4.2. Experimental Investigation of the Burnishing

After the theoretical analysis of the finishing rolling process, it was determined that it is possible to use plastic working by burnishing as a finishing method for thermally sprayed coatings. On this basis, experimental research was carried out on the burnishing process on rolled products with an applied nickel-aluminum alloy and composite matrix on nickel and aluminum with an aluminum oxide dispersion phase.

The burnishing process is a surface plastic working technique that is known by researchers and scientists, but first of all research is carried out on single-layer materials [17,18,19,20,21,22,27,28,29,44,45,46,47,48,49,50,51] and not for coated materials. Therefore, and based on our own research, an experimental analysis of the burnishing of products with the applied alloy and composite coatings was undertaken. For scientific and research purposes, we designed and made a laboratory stand for finishing by finishing rolling based on existing conventional machine tools. A totally new aspect of the laboratory stand was made of a burnisher discoid with a rigid clamp with replaceable burnishing elements with a different rounding radius of the working parts. Burnishing of the outer cylindrical surfaces was executed by means of a NK-01 disc burnisher made at the Department of Marine Maintenance. During burnishing, the following parameters were applied: feed *p* = 0.044 mm/rev–0.8 mm/rev; burnisher pass depth *a_n_* = 0.2 mm–0.3 mm; burnishing force *F* = 0.7 kN–1.1 kN; burnishing speed *v_n_* = 28 m/min; burnishing element with a diameter of ϕ50 mm; and maximum number of machining passes equal to 2. Machine oil was used for lubrication and cooling.

**Figure 11:**
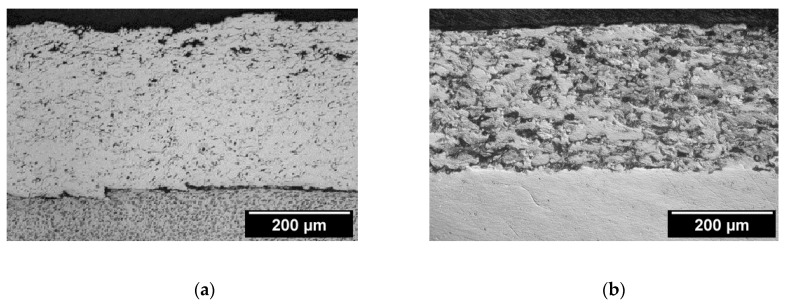
Examples of the microstructure of the Ni–5%Al alloy coating etched with 4% HNO_3_. (**a**) After thermal spraying, the roughness of the coating was Ra = 13.3 μm and (**b**) after burnishing (roller: ϕ50 mm), the roughness of the coating was Ra = 0.2 μm.

In Figure 11, examples of microstructures subjected to surface plastic working and thermal spraying of coatings are shown. The estimated porosity after thermal spraying in alloy coatings was 5%. The porosity after burnishing for the alloy coatings was the same as that obtained.

After experimental tests of the surface plastic working of alloy coatings applied to the steel substrate in [4,13,17], it was determined that there was a 12-multiple decrease of roughness and a strengthening of 25% compared to the machined coatings. The surface roughness after burnishing the alloy coatings was within Ra = 0.2 μm and Ra = 0.3 μm. In connection with the decrease in roughness and surface strengthening, an increase was observed in resistance to corrosion in a sea water environment, contact fatigue, and tribological wear as well as a reduction in the coefficient of friction and obtained a flat characteristic of intensity of wear compared to machining coatings.

**Figure 12:**
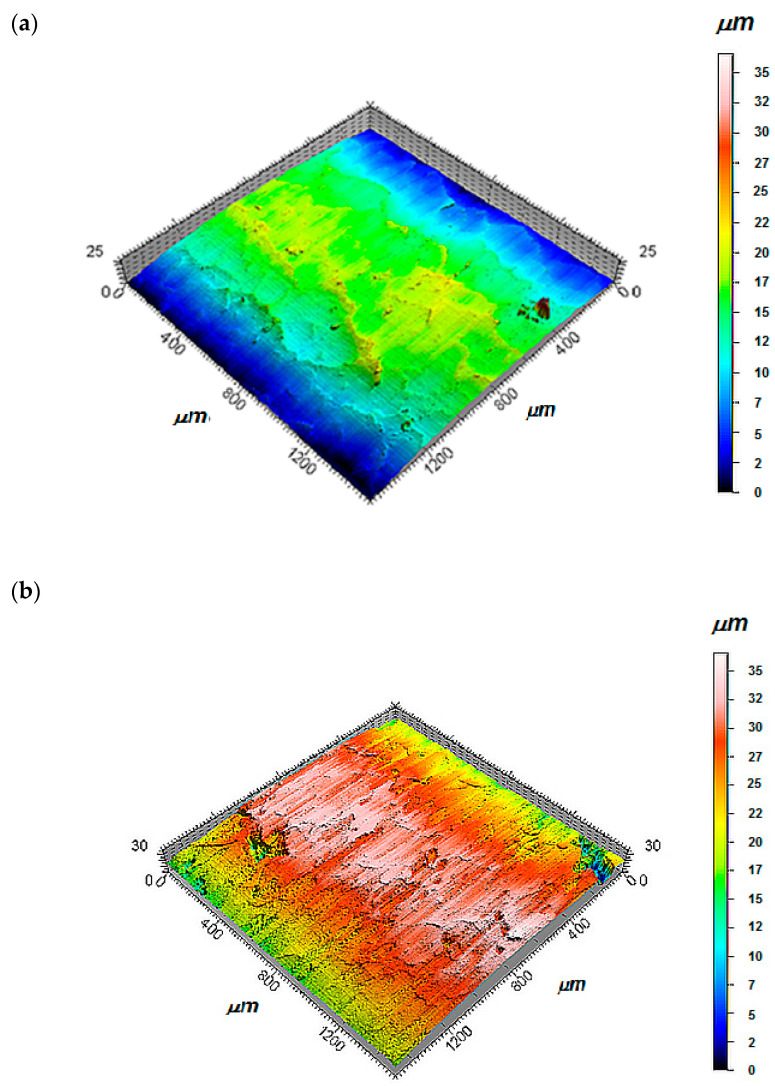
The topography of the burnished surface of the coatings: alloy (**a**) and composite (**b**).

Figure 12 presents sample topographies of the geometric structure of the surface of alloy coatings and composite coatings subjected to finishing burnishing. The surface of the flame spraying alloy coatings obtained as a result of burnishing was characterized by the appearance of folds resulting from the formation before the burnishing element so-called wave, and then the strengthening of the material. These folds compounded according to the tangential velocity vector direction.

**Figure 13:**
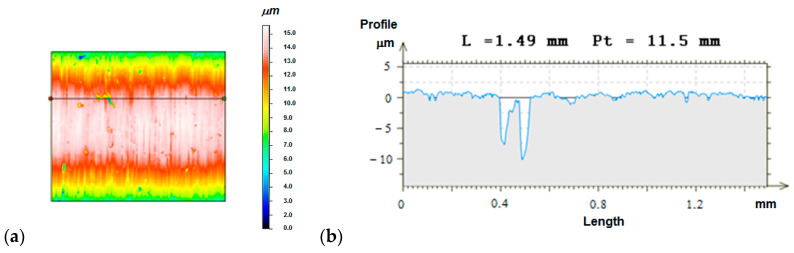
Contour map of the burnished surface of the plasma sprayed composite coatings together with the surface roughness profile. (**a**) topography of the burnished surface, (**b**) surface roughness profile.

Figure 13 shows an example of the roughness profile of the composite coating surfaces together with the contour map after burnishing. Topography was evaluated using a Hommel-Etamic T8000 profilometer. The length of the measuring section was 1.5 mm; step 2.5 mm; number of steps 500; measuring resolution in the longitudinal direction 0.002 μm; and measuring tip radius 2 μm.

After burnishing, it was possible to obtain a surface Ra = 0.2 m–0.3 m of the alloy coating and surfaces Ra = 0.7 m–1.0 m of the composite coatings. Almost a twelve-times decrease in the surface roughness, compared to the turned coatings Ni–5%Al, could be obtained by burnishing with a force of 1100 N; feed 0.08 mm/rev; and speed 28 m/min. In the case of burnishing composite coatings Ni–5%Al–15%Al_2_O_3_ while smooth burnishing and strengthening, it has to use the following parameters of burnishing force of 700 N; feed 0.044 mm/rev; and speed 28 m/min. The number of burnishing tool passes did not fundamentally reduce the roughness and strengthening of the alloy coating Ni–5%Al and composite Ni–5%Al–15%Al_2_O_3_. There was no influence on the type of applied burnish on the average values of roughness, waviness, and material contribution of the burnished coatings Ni–5%Al and Ni–5%Al–15%Al_2_O_3_ obtained by thermal spraying method.

### 4.3. Summary Investigation of the Burnishing and Rolling

The results indicate that there was mostly a reduction in the arithmetic average of the order of roughness profile of the coating surface after burnishing and rolling. After the microhardness measurements were carried out, it was found that after rolling, the microhardness of the tested coatings increased and that the degree of relative strengthening of the S_u_ coating matrix was about 30% larger for a relative draft of 10% than for a draft of 5% (Table 2 and Table 3). The greatest values of strengthening of the Ni–5%Al alloy coating after cold-rolled with a 10% relative draft were obtained. Per degree of relative strengthening of the cold-rolled samples was also affected by the diameter of the working roll. Table 2 and Table 3 show the results of the decrease surface roughness (K_Ra_) and strengthening (S_u_) of the alloy and composite coatings after burnishing with a roller diameter of ϕ50 mm and cold rolling on a mill with a roller diameter of ϕ150 mm and ϕ200 mm.

The coating subjected to plastic working on a rolling mill with a roll diameter of ϕ200 mm obtained a greater value of the degree of relative strengthening compared to the strain coatings with the aid of a roller diameter of ϕ150 mm and after burnishing with a roller diameter of ϕ50 mm.

**Figure 14:**
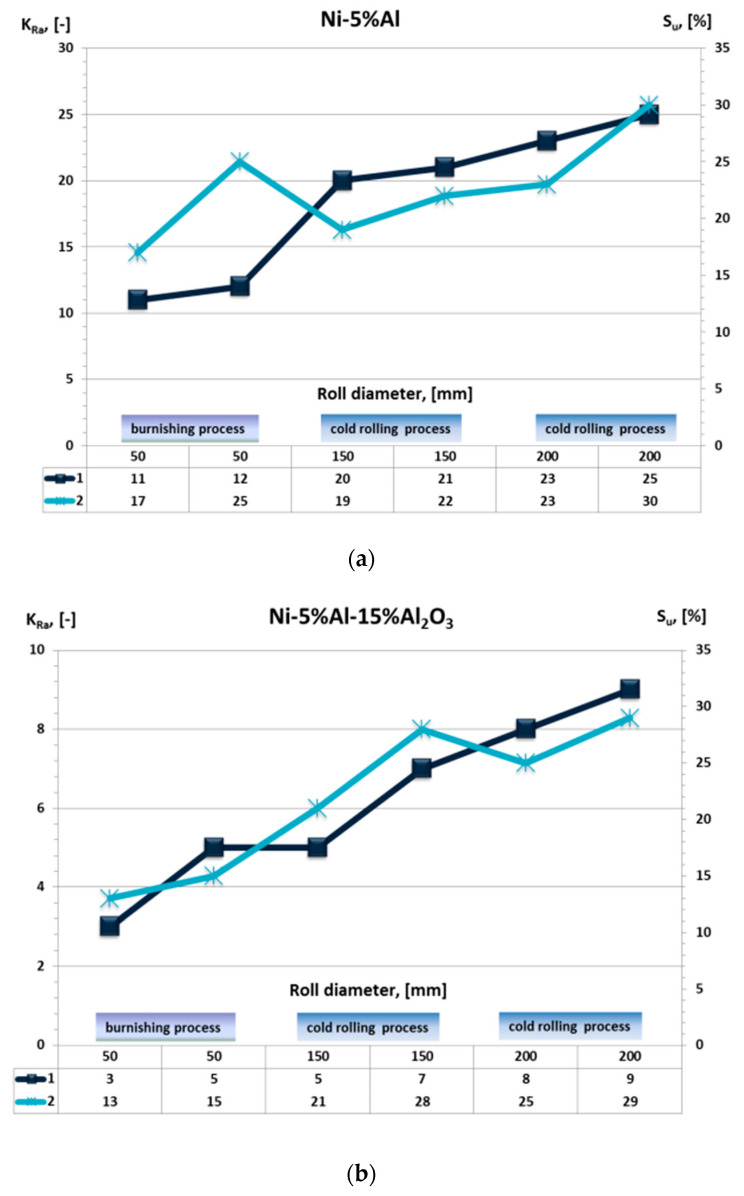
Dependence of the reduction in roughness and increase in strengthening for the (**a**) Ni–5%Al alloy coatings and (**b**) Ni–5%Al–15%Al_2_O_3_ composite coatings, with the diameter of the roller at ϕ50 mm, burnishing; ϕ150 mm, cold rolling; ϕ200 mm, cold rolling; where (1) indicator decrease surface roughness (K_Ra_) and (2) strengthening (S_u_).

Figure 14 shows the results of the value of strengthening and reducing roughness. The alloy and composite coatings achieved a lower roughness and higher hardness after plastic working. It can be seen that for larger roll diameters, higher (K_Ra_) values were obtained and corresponded to the strengthening (S_u_) value. Along with the increase in the diameter of the roller value, the surface layer of coatings was strengthened, which is characteristic of the composite coatings of Ni–5%Al–15%Al_2_O_3_. Using burnishing for finishing the alloy coatings of Ni–5%Al, reinforcement values comparable to those after the cold rolling process can be obtained.

The Ni–5%Al alloy coatings during burnishing were strengthened to 25%. Composite coatings of Ni–5%Al–15%Al_2_O_3_ during burnishing were strengthened to 15% (Figure 14).

Comparing the experimental and numerical studies determined the occurrence of residual stress in the coating material. The stress measurement showed the presence of tensile stresses at the surface after turning and compressive stresses after burnishing. Significant stress anisotropy was observed for the sample after turning. The largest stresses were observed in the axial direction, which amounted to 653 MPa. Axial stresses obtained about 55% higher values than the circumferential stresses. Tangential stresses had an average value of 421 MPa. Burnishing caused a reduction in the tensile internal stress in the surface layer of the alloy coating. The mean value of the main stress in the axial direction after surface forming was 243 MPa, where a 63% reduction in axial stress was found. In the tangential direction, the decrease in the main stress value was smaller. Compared to the turned coatings, 28% less stress was noticed. After burnishing, the average value of peripheral stress was 305 MPa. However, after the numerical analysis of burnishing, the average stress value in the axial direction of 256 MPa and the average circumferential stress value of 322 MPa were determined, making an error of 5%.

In order to strengthen and reduce the roughness of the alloy and composite coatings, the use of plastic working by rolling is recommended. Burnishing can be used to finish only alloy coatings. Burnishing and rolling provides a reduction in roughness and an increase in hardness.

## 5. Summary and Conclusions

Surface plastic working by rolling and burnishing has an advantageous influence on the surface quality of the workpiece, not only by significantly improving the roughness, but also by increasing the strength properties of the surface layers. Plastic working of the surface layer is more economical and ecological compared to abrasive machining.

Products with alloy and composite coatings may be used for elements in marine equipment and mechanical machines (e.g., centrifugal pumps). Machine parts with coatings after plastic working can obtain adequate strength properties and performance to work in a marine environment and they can be used in the shipbuilding industry. Thermal spray coatings based on nickel can also be used to regenerate shaft necks of marine centrifugal pumps for pumping sea water. An important advantage of the technology of flame coating is its simplicity as it can be successfully carried out by crew members or repair crews, even during the voyage. As a finish of these coatings, surface plastic working in exchange for abrasive machining can be used.

The main purpose of this article was to emphasize the use of plastic working process (rolling and burnishing) as (surface) coating finishing, for instance, in ship pump shaft regeneration.

Due to the technological and economic aspects, on the basis of numerical analysis of the cold working process by rolling flat products with a coating applied by flame spraying, the optimal thickness of the nickel-based coating was selected, which was equal to h_p_ = 0.6 mm. Increasing both the thickness of the coating and the draft caused a disadvantageous distribution of the substitute strains, which could lead to delamination of the band. Therefore, it is best to apply thin coatings and use small drafts.

After performing model tests of the rolling process, we determined that it is possible to use plastic working as a finishing technique of thermally sprayed coatings.

For economic reasons, it can be concluded that finishing in experimental tests of cold-rolling of thermally sprayed coatings did not cause material losses.

- After cold rolling on a rolling mill with a roller diameter of ϕ150 mm, we obtained the surface roughness of the Ni–5%Al alloy coatings of Ra = 0.49 m for the 10% draft, and Ra = 0.52 m for the 5% draft. For cold-rolled samples on a rolling mill with a roller diameter of ϕ200 mm, the coatings showed less roughness (Ra = 0.28 µm for 10% draft, Ra = 0.33 m for 5% draft).
- The roughness of the coatings after plastic working decreased by 25-times. It was found that there was a 30% strengthening of the matrix alloy coatings.
- The surface roughness of the burnished alloy coatings, irrespective of spraying technology and pre-treatment parameters, were contained in an arithmetic mean range of the ordinate of the profile Ra = 0.2 m–0.3 m. In the case of the composite coatings, Ra = 0.7 m–1.0 m was the result.
- As a consequence of surface plastic working, there was a 12-times reduction in roughness and 25% strengthening in the alloy coatings.
- The porosity after the burnished coatings was reduced.

As a result of the numerical and experimental cold rolling and burnishing tests, it was concluded that there was a significant reduction in the porosity of the plastically deformed coating, a low surface roughness was achieved, and the coating hardness increased after plastic working.

The analysis of the research confirmed that plastic working can be used in the shipbuilding industry to develop the selected flame sprayed coating parameters for alloys and composite coatings.

## Figures and Tables

**Table 1:** The mechanical properties of C45 unalloyed steel and chemical composition in weight %.

C	Cr	Mo	Mn	Si	Ni	P	S
0.42–0.5	0.4	0.1	0.5–0.8	0.1–0.4	0.4	0.045	0.045
R_e_ [MPa]	R_m_ [MPa]	A [%]	Z [%]	HB	KV [J]		
275–490	600–850	14–17	35–45	229	25		

**Table 2:** The indicator decrease in surface roughness (K_Ra_) and strengthening (S_u_) of the Ni–5%Al alloy coatings after burnishing and rolling.

Roll Diameter [mm]	Relative Draft [%]	Rz [μm]	Ra [μm]	K_Ra_ [–]	HV	S_u_ [%]
50	0.5	2.38	0.20	11	213	17
50	1.0	2.77	0.30	12	225	25
150	5.0	4.42	0.52	20	215	19
150	10.0	4.48	0.49	21	222	22
200	5.0	2.86	0.33	23	212	23
200	10	2.48	0.28	25	234	30

**Table 3:** The indicator decrease in surface roughness (K_Ra_) and strengthening (S_u_) of the Ni–5%Al–15%Al_2_O_3_ composite coatings after burnishing and rolling.

Roll Diameter [mm]	Relative Draft [%]	Rz [μm]	Ra [μm]	K_Ra_ [–]	HV	S_u_ [%]
50	0.5	4.88	0.70	3	209	13
50	1.0	5.66	1.00	5	211	15
150	5.0	14.37	2.69	5	217	21
150	10.0	11.59	2.02	7	231	28
200	5.0	9.61	1.84	8	225	25
200	10.0	8.34	1.52	9	237	29
